# Transthyretin promotes the invasion of combined hepatocellular cholangiocarcinoma by tumor‐associated macrophages

**DOI:** 10.1002/cnr2.1888

**Published:** 2023-09-09

**Authors:** Kun Ke, Junqing Lin, Ning Huang, Leye Yan, Rihua Liao, Weizhu Yang

**Affiliations:** ^1^ Department of Interventional Radiology Fujian Medical University Union Hospital Fuzhou China

**Keywords:** AKT, combined hepatocellular cholangiocarcinoma, ERK, TAMs, transthyretin

## Abstract

**Background:**

Patients with combined hepatocellular‐cholangiocarcinoma (cHCC‐CCA) have limited treatment options and poor prognosis. Tumor‐associated macrophages (TAMs) are the most abundant infiltrating immune cells in the tumor microenvironment and promote tumor stemness, proliferation, invasion and metastasis. Evidence suggested that transthyretin (TTR) influenced the prolifetation and invasion functions of different tumors and play an essential role in the tumor microenvironment.

**Aims:**

To investigate the involvement of TTR in TAMs affecting the invasion of cHCC‐CCA.

**Methods and Results:**

Data sets obtained from the Gene Expression Omnibus database were integrated. Differentially expressed genes (DEGs) were obtained using R software, and modules associated with cHCC‐CCA were screened by weighted gene co‐expression network analysis (WGCNA). Human THP‐1 cells were induced to differentiate into macrophages and then co‐cultured with HCCC9810 cells and tumor necrosis factor‐α (TNF‐α) to simulate the inflammatory microenvironment of cHCC‐CAA. In addition, small interfering RNA against TTR was transfected into HCCC9810 cells, and recombinant TTR and ERK and AKT‐specific inhibitors were added to HCCC9810 cells, respectively; after that, the levels of NF‐κB protein and phosphorylated ERK and AKT were measured. The invasive abilities of HCCC9810 cells were also tested.

One hundred forty‐five DEGs were associated with cHCC‐CCA, of which TTR was up‐regulated. Turquoise modules containing TTR in WGCNA were most significantly associated with cHCC‐CCA. TTR was highly expressed in HCCC9810 compared to Huh‐28. HCCC9810 showed enhanced invasive capacity after co‐culture with TNF‐α + macrophages (*p* < .05). After interfering with TTR, the invasive ability of HCCC9810 was diminished, accompanied by decreased expression of NF‐κB, p‐ERK1/2, and p‐AKT (*p* < .05). After treating HCCC9810 with ERK and AKT‐specific inhibitors, the invasive ability of HCCC9810 was diminished, accompanied by decreased expression of NF‐κB and TTR (*p* < .05).

**Conclusion:**

TTR can promote the invasive ability of cHCC‐CCA by regulating AKT/NF‐κB and ERK pathways with the assistance of TAMs.

## 1 BACKGROUND

Primary hepatocellular carcinomas are mainly classified as hepatocellular carcinoma (HCC), intrahepatic cholangiocarcinoma (iCCA), and combined hepatocellular‐cholangiocarcinoma (cHCC‐CCA). cHCC‐CCA is a rare subtype of primary liver malignancy with an incidence of 0.4–14.2%, varying from report to report.^1^, ^2^ Histologically, cHCC‐CCA has two distinct morphological features, including HCC and iCCA.^3^ Surgical resection, including lymph node dissection, remains the only option for patients with cHCC‐CCA to achieve a cure.^4^ However, tumor recurrence is frequent (up to 80% within 5 years), and the 5‐year survival rate does not exceed 30%.^5^ Most patients are diagnosed at an intermediate to an advanced stage and are lost to surgical treatment. There are few studies on systematically treating unresectable cHCC‐CCA.^6^, ^7^, ^8^ Sorafenib and chemotherapy, which are treatments for HCC and iCCA, do not provide significant benefits to unresectable cHCC‐CCA.^9^ Therefore, searching for molecules that impact the biological behavior of cHCC‐CCA may provide new ideas for treating unresectable cHCC‐CCA.

Tumor‐associated macrophages (TAMs) are the most abundant infiltrating immune cells in the tumor microenvironment, producing chemokines that promote tumor stemness, proliferation, invasiveness, and metastasis.^10^ TAMs can be classified into M1 and M2 types according to their functions, with the M2 phenotype predominating. Activated M2‐like macrophages can secrete cytokine CCL22, which enhances tumor invasion and induces epithelial‐mesenchymal transition (EMT) by activating Smad and up‐regulating Snail.^11^ In vitro experimental data show that M2‐TAMs promote tumor development by secreting various cytokines, such as tumor necrosis factor‐α (TNF‐α), ICAM‐1, and IL‐6, and regulating the EMT of cancer cells, thereby coordinating the immune microenvironment of iCCA.^12^ The primary biological function of M1 macrophages is to kill tumor cells and pathogens. However, M1‐like TAMs have also shown a positive association with cancer.IL‐1β secreted by M1 macrophages induces the expression of programmed cell death protein ligand 1 (PD‐L1) through the transcription factors IRF1 and NF‐κB, activating hepatocellular carcinoma cells.^13^ Recently, it has been found that infiltration of M1‐like TAMs is associated with the aggressive characteristics of the tumor.^14^, ^15^ M1‐like TAMs may play an essential role in the development of tumors.

Transthyretin (TTR), a tetrameric protein of 127 amino acids, is a carrier protein for thyroxine and retinol. TTR can be used as a potential tumor marker to predict the prognosis of tumors such as pancreatic ductal adenocarcinoma^16^ and renal cell carcinoma.^17^ It has been reported that TTR can regulate MAPK/ERK signaling pathways to promote drug resistance, proliferation, and metastasis in prostate cancer cells.^18^ Another study has found that TTR promotes the proliferation and growth of lung tumor cells in lung cancer through activation of the AKT/mTOR and NF‐κB signaling pathways. At the same time, TTR acts as a cytokine to influence the differentiation function of bone marrow cells and plays a role in the tumor microenvironment.^19^ Some studies have detected changes in TTR expression in the serum of CCA patients by proteomics,^20^ but the exact mechanism has not been further elucidated. Moreover, there are no studies on TTR and cHCC‐CCA.

This study investigated the relationship between TTR and cHCC‐CCA through bioinformatics and cellular experiments. To investigate the effect of TTR on the invasive ability of cHCC‐CCA and the interaction between tumor cells and TAMs, and to explore the signaling pathways involved in this process, to provide new therapeutic ideas and molecular targets for the systemic treatment of unresectable cHCC‐CCA.

## 2 MATERIALS AND METHODS

### 2.1 Data collection

All three data sets (GSE15765, GSE35306, GSE32879) were obtained from the Gene Expression Omnibus (GEO) database. GSE15765, a study of cholangiocarcinoma‐like gene expression traits in hepatocellular carcinoma, contains 70 HCC, 13 CCA, and 7 cHCC‐CCA. GSE35306, a survey on the progenitor features and signaling pathways in combined hepatocellular‐cholangiocarcinoma contains 3 CCA, 7 HCC, and 20 cHCC‐CCA. GSE32879 deals with the hepatic stem‐like phenotype and the interaction between EMT and miR‐200c in intrahepatic cholangiocarcinoma, has 16 CCA, 7 cHCC‐CCA, 2 hepatic adenomas, 5 focal nodular proliferations, and 7 non‐tumor tissues. For the above study, we only extracted mRNA data from CCA and cHCC‐CCA samples for inclusion.

The transcriptome data were log2 transformed, unnormalized, and then used for subsequent analysis. Due to the small sample size, the “sva” R package (version 4.2.1) was used to combine these three data sets (GSE15765, GSE35306, GSE32879) into a combined data set of 32 CCAs and 34 cHCC‐CCAs, and this integrated data set was batch correction. The merged data set was screened for differentially expressed genes (DEGs) using the “limma” R package. |Log2 fold change| > 1 and adj.*p* < :05 were set as the thresholds for screening DEGs. In addition, volcano plots of DEGs were constructed by the “ggplot2” R package. The online tool imageGP constructed heatmaps and box plots of TTR expression.^21^

### 2.2 Weighted correlation network analysis

The weighted gene co‐expression network analysis (“WGCNA”) R package was used to construct co‐expression networks for cHCC‐CCA and CCA and to analyze modules significantly associated with the cHCC‐CCA phenotype. First, the processed data were imported into WGCNA, and only genes with more than 25% variation were retained. Second, samples were stratified and clustered to exclude outliers, ensuring the reliability of network construction. Third, a pick‐soft‐threshold function was used to derive a soft‐threshold power β by co‐expressing similarity and calculating the neighborhood degree, which was transformed into a topological overlap matrix (TOM). Fourth, the mean linkage hierarchical clustering based on the dissimilarity measure of the TOM was used to construct a gene dendrogram with a minimum gene dendrogram size of 30. Fifth, module membership (MM) and gene significance (GS) were calculated for modules related to clinical attributes to filter essential genes of candidate modules. The screened DEGs and genes in the key modules were intersected using the “VennDiagram” R package. Gene Ontology (GO) and Kyoto Genome Encyclopedia (KEGG) pathway analyses were performed on the intersected genes using the “clusterProfiler” R package. Thresholds for analysis were set at counts >2 and adjusted *p* < .05.

### 2.3 Cell culture

Human cholangiocarcinoma cells Huh‐28 (BNCC339776, BeNa Culture Collection) were cultured in a DMEM medium (Hyclone, KGM12800S, KeyGEN BioTECH). Human hepatic cholangiocarcinoma cells HCCC9810 (BNCC351917, BeNa Culture Collection) were cultured in RPMI 1640 medium (Hyclone, kgm31800, KeyGEN BioTECH). Human intrahepatic bile duct epithelial (IBE) cells (HUM‐iCell‐d014, Sebacom) were cultured in primary epithelial cell medium (PriMed‐iCell‐001, Sebacon) at 37°C, 5% CO2, 95% humidified air. The above medium was supplemented with 10% fetal bovine serum, penicillin 80 U/mL, and streptomycin 0.08 mg/mL. Human monocyte THP‐1 (BNCC341989, BeNa Culture Collection) was resuspended in an induction medium containing 100 ng/mL PMA (P6741, Solarbio) for 48 h, stimulated to differentiate into macrophages and then transferred to cancer cells for 24 h.

### 2.4 CCK‐8

Tumor cells were first added with different concentrations of TNF‐α (C008, Novoprotein) and incubated under the above culture conditions for 24 h. After digestion with trypsin, the cells were treated with 10% FBS medium to prepare single‐cell suspensions. The resulting cell suspension was centrifuged at 2500 rpm for 3 min; 1 mL of PBS was added and centrifuged again for 3 min. CCK‐8 (KGA317, KeyGEN BioTECH) was added to 96‐well plates at 10 μL per well and incubated at 37°C for 4 h. The optical density of each well was detected at 450 nm using an enzyme marker. The TNF‐α concentration (100 ng/L) was determined according to the cell viability and relevant references (Figure S1).

**FIGURE 1:**
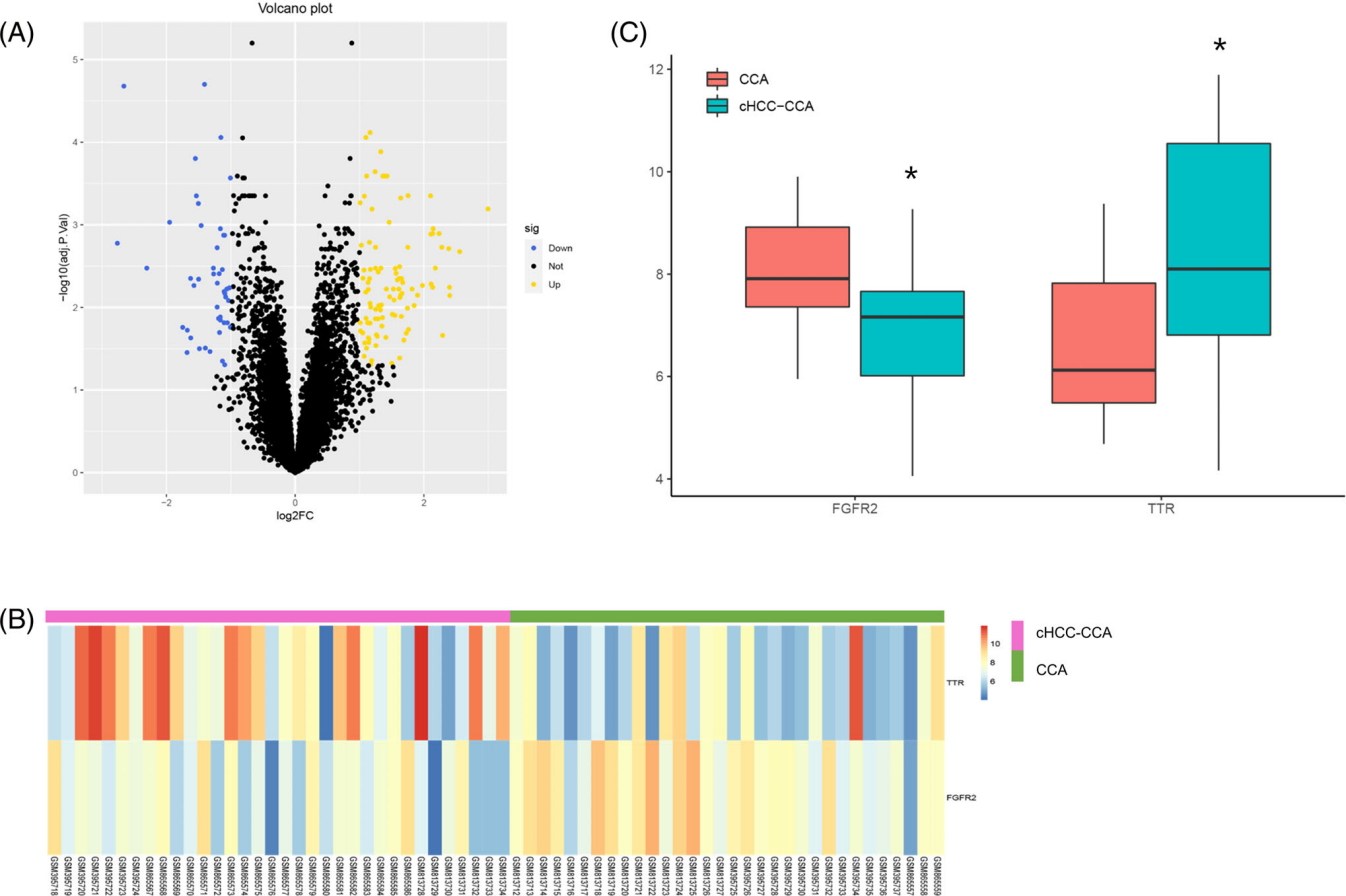
Bioinformatics analysis of cHCC‐CCA and CCA. (A) Volcano plot of DEGs, yellow plot points represent up‐regulated DEGs, and blue plot points show downregulated DEGs. (B) Heatmap of TTR and FGFR2 expression. (C) Box plot of TTR and FGFR2 expression in cHCC‐CCA and CCA groups. *indicates compared with the CCA group, *p* < .05.

### 2.5 Grouping

First, tumor cells were grouped as control (HCCC9810 cells), HCCC9810 cells + TNF‐α (100 ng/L), and HCCC9810 cells + macrophages + TNF‐α. Second, after silencing TTR and adding recombinant TTR, HCCC9810 cells were divided into four groups as follows: (1) HCCC9810 cells + macrophages + TNF‐α (control), (2) HCCC9810 cells + siRNA TTR (siTTR) + macrophages + TNF‐α, (3) HCCC9810 cells + siRNA negative control (siNC) + macrophages + TNF‐α, and (4) HCCC9810 cells + recombinant TTR (rTTR, 1 μM, C545, Novoprotein) + macrophages + TNF‐α group. Finally, LY3214996 (HY‐101494, MCE) and MK‐2206 (2HCL) (HY‐10358, MCE) were added to the HCCC9810 cells + TNF‐α group, respectively, and the related protein expression was detected after blocking the ERK and AKT pathways.

### 2.6 TTR interference vector construction

The NCBI search for TTR accession numbers and their sequences set up three TTR knockdown siRNA inserts with the template sequences, siTTR‐1: (forward primer) 5′‐GGAUAUACAAAGUGGAAAUTT‐3′ and (reverse primer) 5′‐AUUUCCACUUUUGUAUAUCCTT‐3′; siTTR‐2: (forward primer) 5′‐GGUCAAAGUUCUAGAUGCUTT‐3′, and (reverse primer) 5′‐AGCAUCUAGAACUUUGACCTT‐3′; siTTR‐3: (forward primer) 5′‐GCCAUUUGCCUCUGGGAAATT‐3′ and (reverse primer) 5′‐UUUCCCAGAGGCAAAUGGCTT‐3′; negative control sequences were (forward primer) 5′‐UUCUCCGAACGUGUCACGUTT‐3′, (reverse primer) 5′‐ACGUGACACGUUCGGAGAATT‐3′. Biosystems (Anhui) Co synthesized these oligonucleotides. The recombinant TRR‐siRNA vector and the negative control vector were transfected into HCCC9810 cells using Lipofectamine 3000 (Invitrogen, L3000015) according to the instructions, and one of the siRNAs was selected for subsequent experiments by Western blot to detect TTR protein expression levels.

### 2.7 Fluorescent quantitative PCR (RT‐qPCR)

Each group of cells was taken for RNA extraction. mRNA extraction was carried out using the mRNA ultra‐pure extraction kit. mRNA concentration and purity (OD260/OD280) were determined using a UV–visible spectrophotometer. cDNA was synthesized by the mRNA reverse transcription kit, respectively. Fluorescent quantitative PCR was carried out using a fluorescent PCR instrument, with β‐actin as an internal reference and the relative expression of genes. The amount was calculated according to the 2‐ΔΔCt method. The TTR primer sequences were as follows: (forward primer) 5′‐TGGGAGCCATTTGCCTCTG‐3′ and (reverse primer) 5′‐ AGCCGTGGTGGAATAGGAGTA‐3′ for TTR; (forward primer) 5′‐TGGCACCCAGCACAATGAA‐3′ and (reverse primer) 5′‐CTAAGTCATAGTCCGCCTAGAAGCA‐3′ for β‐actin.

### 2.8 Western blot assay

Cell samples were lysed with RIPA lysate (C1053, Beijing Pulley Gene Technology Co., Ltd.) containing 10% PMSF (Elabscience) and Na3VO4 (Elabscience) at four‐degree centigrade for ~20 min, and sample cells were sufficiently fragmented to extract proteins using a cell disruptor. The lysate was centrifuged at 12000 rpm and 4°C for 10 min, and the supernatant was collected. The amount of protein in each set of supernatants was quantified using the BCA Protein Assay Kit (E‐BC‐K318‐M, Elabscience). Each set of supernatants was then boiled for 5 min with a loading buffer, and 10 μg of total protein was loaded onto SDS polyacrylamide and separated by electrophoresis. The separated protein bands were transferred to polyvinylidene difluoride (PVDF) (IPVH00010, Millipore) membranes. The membranes were incubated with the following antibodies: anti‐TTR, 1:1000 from proteintech; anti‐NF‐KB, 1:1000 from CST; anti‐p‐ERK, 1:1000 from Affinity; and anti‐p‐AKT, 1:1000 from Affinity. Next, the membranes were incubated with TBST (10 mL of 1 mol/L Tris‐HCL, pH 7.5, 8.8 g NaCl, and 1 mL Tween‐20, the total volume of 1 liter) three times (15 min per rinse), followed by incubation with anti‐rabbit or anti‐mouse secondary antibody (1:2000) for 60 min at room temperature. After that, the membranes were rinsed three times (15 min each) with TBST. Finally, protein bands on membranes were visualized by enhanced chemiluminescence. Finally, protein bands on membranes were visualized by enhanced chemiluminescence (ECL) reagent (RJ239676, Thermo Fisher).

### 2.9 Transwell assay

HCCC9810 cells were resuspended in RPIM‐1640 without FBS at a concentration of 2 × 10^5^ cells/mL; 300 μL of cells were inoculated into each upper chamber, and 500 μL of RPIM‐1640 containing 20% FBS was added to each lower layer. After incubation at 37°C for 24 h, the medium in the upper chamber was removed, and the cells were wiped with a cotton swab. The chambers were washed twice with PBS, and the remaining cells were removed. The remaining cells were wiped with methanol at 4°C for 10 min. At room temperature, the cells were stained with 0.1% crystal violet for 1 h. Count the number of cells under a light microscope (BX43, Olympus). After the photographing was completed, the staining solution was removed and treated with 33% acetic acid, and the absorbance value of each well was measured at 562 nm using an enzyme marker (WD‐2012B, Beijing Liuyi Biotechnology Co., Ltd.).

### 2.10 Wound‐healing assay

HCCC9810 was inoculated in a 24‐well plate, and the cells were scribed after the cell density reached 90% or more. A 200 μL gun was used to scratch in each well, and the medium was discarded and washed three times with PBS and replaced with the serum‐free medium before taking pictures of the scratches in each well; the cells were placed in the incubator, and the scratches in each well were photographed again after 24 h; the width of the scratches was measured, and the migration rate of the cells was then calculated.

### 2.11 Statistical methods

All experiments were technically repeated three times. GraphPad Prism 9.0 software was applied for statistical analysis and graphing. Quantitative results were expressed as mean ± standard deviation (mean ± SD). One‐way ANOVA was used to compare quantitative values between multiple groups, with *p* < .05 indicating significant differences.

## 3 RESULTS

By analyzing the combined data sets, we obtained 145 DEGs, of which 99 were up‐regulated and 46 were down‐regulated. The results of the DEGs were visualized as volcano plots and heat maps (Figure 1A,B). TTR expression was significantly higher in the cHCC‐CCA group compared to the CCA group (logFC = 1.74, adj.*p* = .010, Figure 1C). In contrast, recombinant fibroblast growth factor receptor 2 (*FGFR2*) was significantly higher in the CCA group (logFC = 1.05, adj.*p* = .015).

**FIGURE 2:**
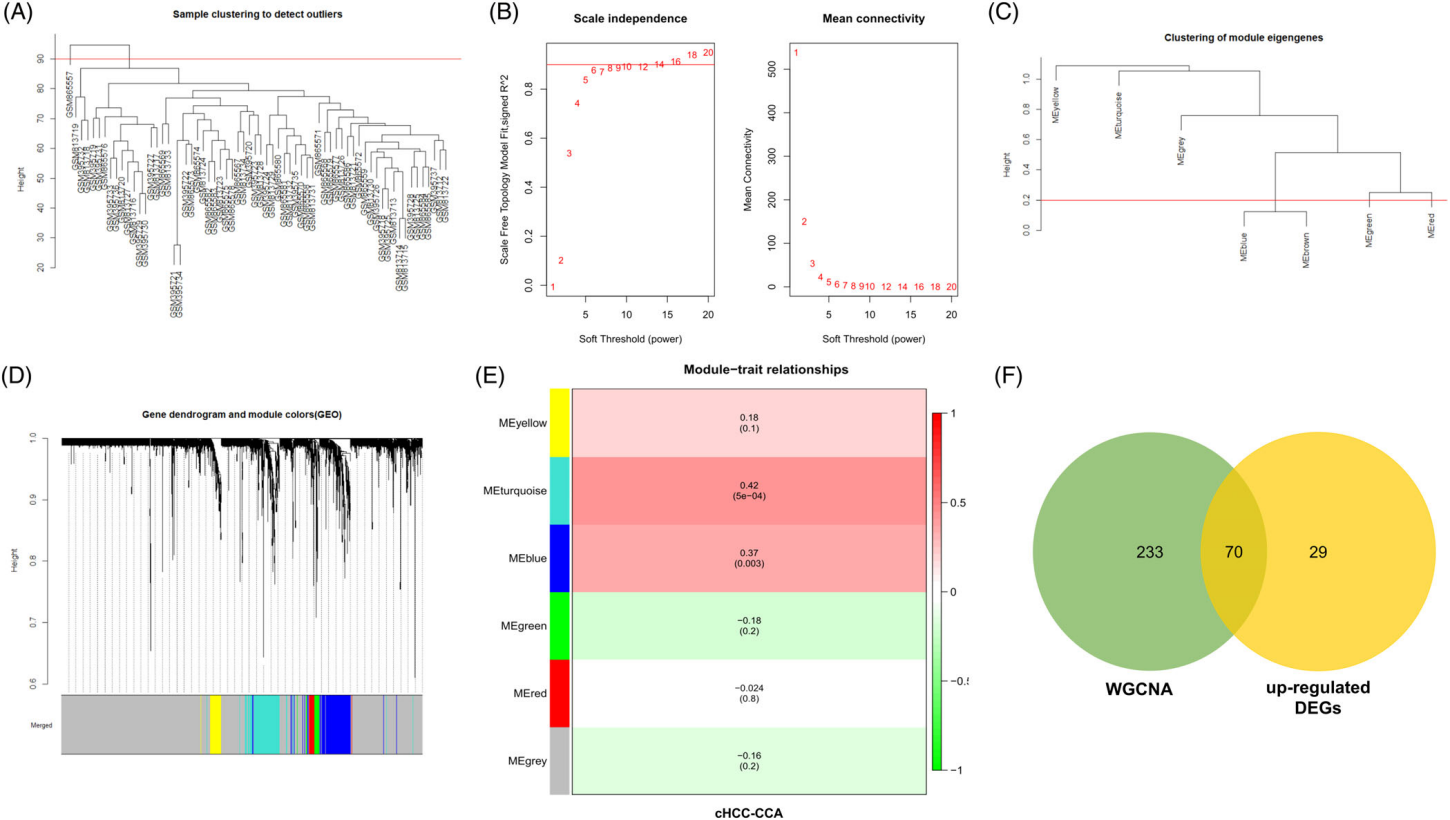
The results of weighted gene co‐expression network analysis (WGCNA). (A) Sample clustering to detect outliers. One outlier sample was removed. (B) The relationship of soft threshold and scale independence or mean connectivity. (C) Clustering of module eigengenes. Two similar modules were merged (threshold 0.2). (D) Gene dendrogram and module colors. (E) Heat map of the association between modules and cHCC‐CCA. (F) Venn diagram showing overlapping genes between the up‐regulated DEGs and the genes in the turquoise module.

We performed WGCNA on the merged data set. Based on the sample clustering tree (Figure 2A), the threshold was set to 90, and one outlier sample was removed. A scale‐free network was constructed, with an optimal soft threshold of β = 14 when the scale‐free R^2^ equals .9 (Figure 2B). Next, we made gene co‐expression modules using hierarchical clustering and dynamic tree clipping. We merged similar modules in the clustering tree, setting the threshold to 0.2 to obtain six modules with genes having similar co‐expression characteristics (Figure 2C). We then calculated correlations between module membership (correlations between specific genes and module eigengene) and gene importance (correlations between specific genes and clinical variables) for each module (Figure 2E). We found that the turquoise module had the most significant correlation with cHCC‐CCA (*r* = .42; *p* = 5e−04) and that the TTR was located in the turquoise module. Therefore, 303 genes in the turquoise module were retained for subsequent analysis.

We took the intersection of the screened up‐regulated genes with those in the turquoise module to obtain 70 overlapping up‐regulated genes (Figure 2F). These overlapping genes were subjected to GO and KEGG analysis. Functional enrichment analysis showed that the overlapping up‐regulated genes were mainly involved in biological processes such as “regulation of blood coagulation,” “cholesterol and lipoprotein metabolism,” and “tyrosine metabolism” (Tables S1 and S2).

**FIGURE 3:**
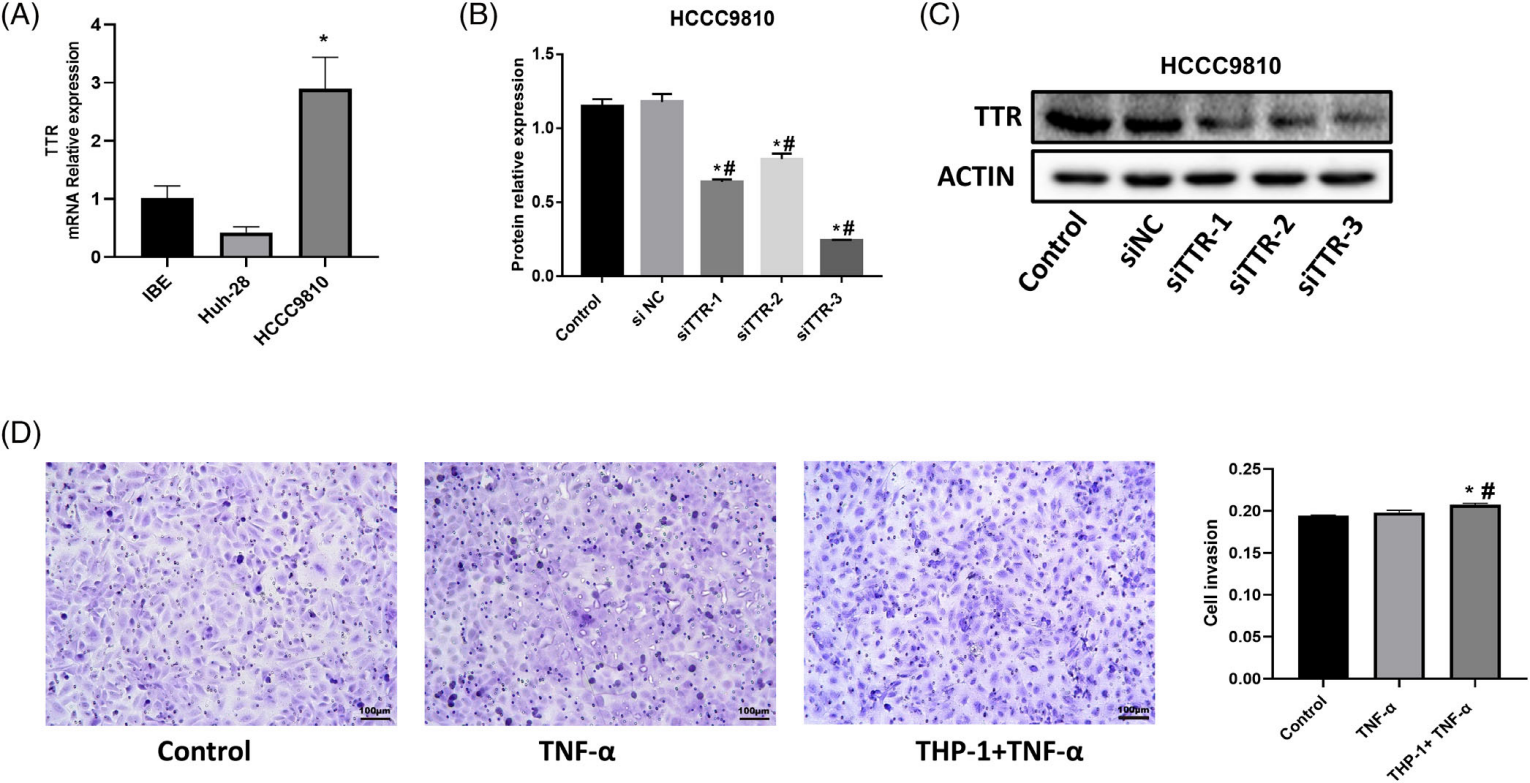
TTR expression and invasive ability of HCCC9810. (A) RT‐qPCR detection of relative TTR mRNA expression in IBE, Huh‐28, and HCCC9810 (*p < .05 vs. IBE). (B) and (C) Three different siRNAs interfere with relative TTR protein expression in HCCC9810 (*p < .05 vs. control, #*p* < .05 vs. siNC). (D) Transwell assay was performed to detect the invasion of HCCC9810 cells. Invasion of HCCC9810 cells receiving different treatments. HCCC9810 cells were co‐cultured with macrophages for 24 h. (*p < .05 vs. Control, #*p* < .05 vs. TNF‐α).

As shown in Figure 3A, it can be seen that TTR was significantly highly expressed in HCCC9810 compared with IBE. Interference with TTR by siRNA showed that TTR expression was significantly lower in the siTTR‐1/2/3 groups compared with the Control and siRNA NC groups. The results were most significant in the siTTR‐3 group (*p* < .05, Figure 3B,C). Therefore, siTTR‐3 was selected for follow‐up experiments.

After co‐culture of HCCC9810 with THP‐1 + TNF‐α, the invasive ability of the cells was examined. The results showed that the invasive ability of HCCC9810 was significantly increased in the THP‐1 + TNF‐α group compared to the blank cells and TNF‐α group (*p* < .05, Figure 3D). This result indicates that the microenvironment constructed by TNF‐α + THP‐1 significantly promoted the invasion of HCCC9810.

**FIGURE 4:**
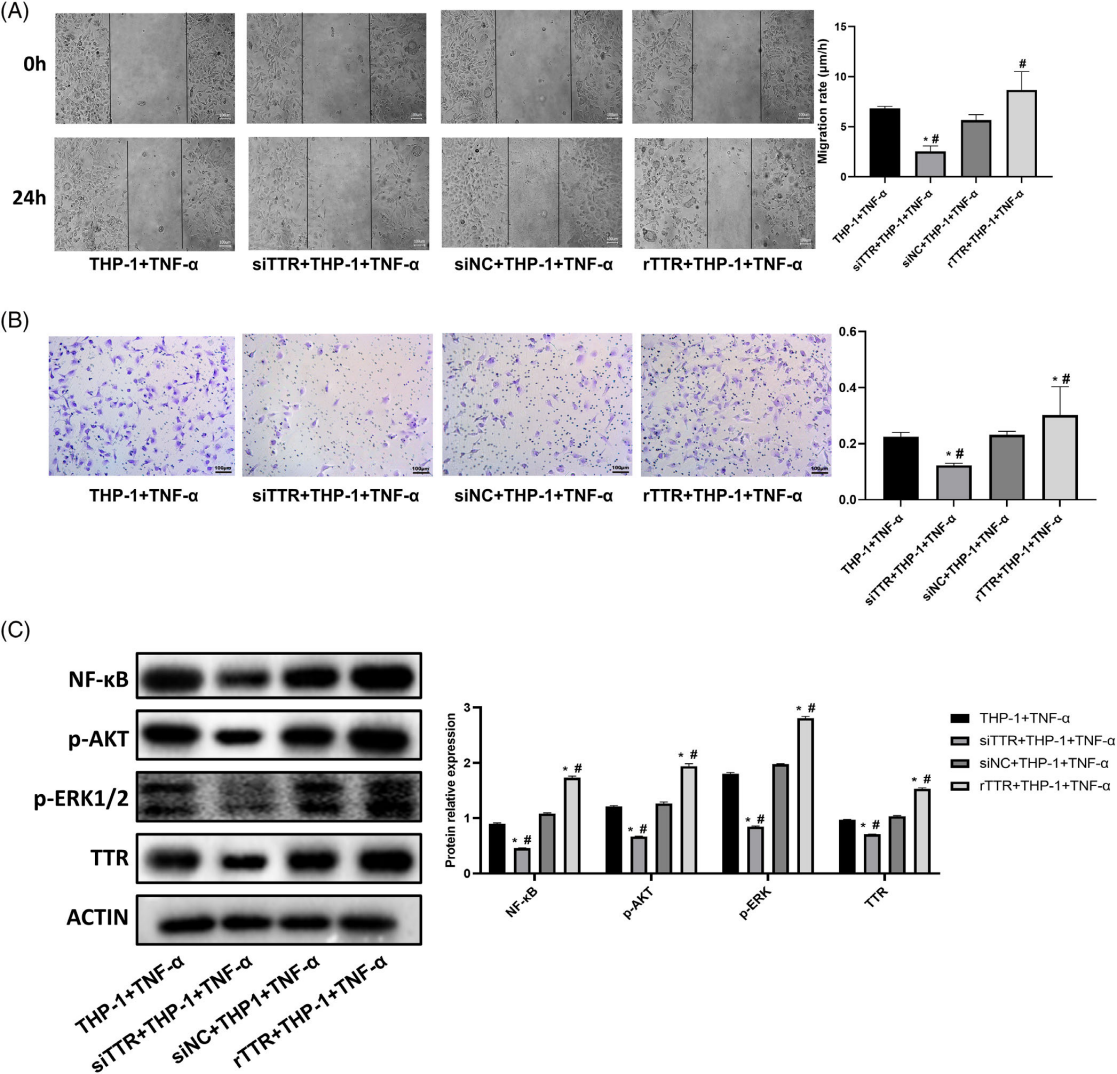
TTR affects the invasion and migration of HCCC9810 cells through macrophages. (A) Migration ability of HCCC9810 cells after TTR silencing and addition of recombinant TTR, respectively. (B) Invasion ability of HCCC9810 cells after TTR silencing and addition of recombinant TTR, respectively. (C) Expression and phosphorylation of NF‐κB, AKT, ERK, and TTR‐related proteins in HCCC9810 cells after TTR silencing and addition of recombinant TTR, respectively. (*p < .05 vs. THP‐1 + TNF‐α. #*p* < .05 vs. siNC+THP‐1 + TNF‐α).

After interfering with the TTR expression of HCCC9810, the tumor cells were co‐cultured with THP‐1 + TNF‐α. The results showed that the migration and invasion ability of HCCC9810 cells in the siTTR+THP‐1 + TNF‐α group was significantly reduced compared with the THP‐1 + TNF‐α and siNC+THP‐1 + TNF‐α groups (*p* < .05), as shown in Figure 4A,B. The migratory and invasive ability of HCCC9810 cells in the TTR + THP‐1 + TNF‐α group was significantly increased compared with the siNC+THP‐1 + TNF‐α group (*p* < .05). This result indicates that TTR promoted the migration and invasion ability of HCCC9810 cells under the combined effect of low‐dose TNF‐α and macrophages.

As shown in Figure 4C, the expression of NF‐κB, p‐ERK, p‐AKT, and TTR proteins was significantly decreased in HCCC9810 cells in the siTTR+THP‐1 + TNF‐α group compared with the THP‐1 + TNF‐α and siNC+THP‐1 + TNF‐α groups (*p* < .05). Compared with the THP‐1 + TNF‐α and siNC+THP‐1 + TNFα groups, NF‐κB, p‐ERK, p‐AKT, TTR protein expression was significantly increased in HCCC9810 cells in the rTTR+THP‐1 + TNFα group (*p* < .05).

**FIGURE 5:**
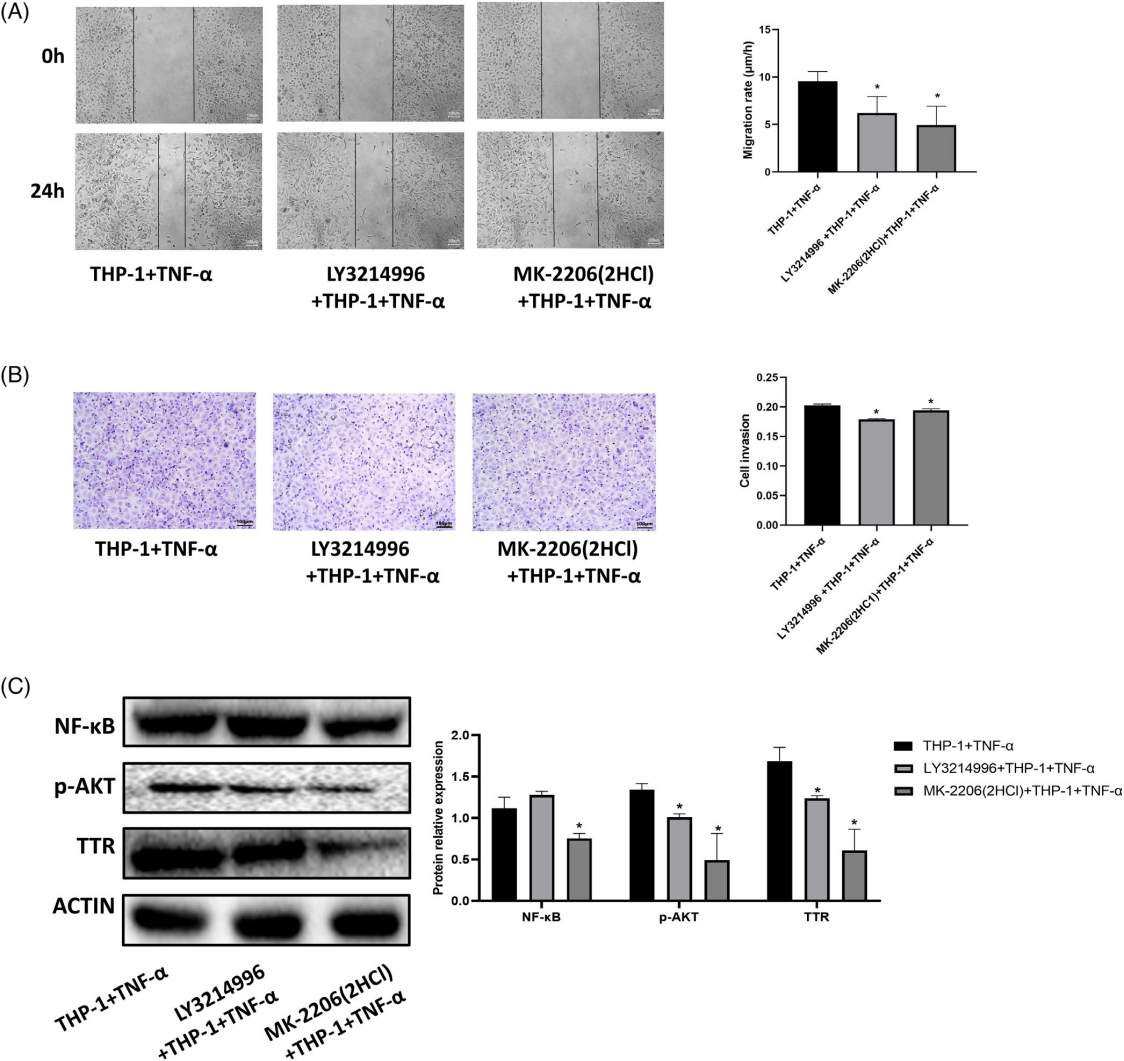
Blocking the AKT/NF‐κB and ERK pathways inhibited the invasion and migration of HCCC9810 cells. (A) Migratory ability of HCCC9810 cells treated with LY3214996 and MK‐2206(2HCl) for 24 h. (B) Invasion ability of HCCC9810 cells treated with LY3214996 and MK‐2206(2HCl) for 24 h. (C) Invasion ability of HCCC9810 cells treated with LY3214996 and MK‐2206(2HCl) for 24 h. expression and phosphorylation of NF‐κB, AKT, and TTR‐related proteins in HCCC9810 cells. (*p < .05 vs. THP‐1 + TNF‐α). ly3214996: ERK inhibitor, MK‐2206(2HCl): AKT inhibitor.

Figure 5A,B show the migration and invasion ability of HCCC9810 in the LY3214996 + THP‐1 + TNF‐α and MK‐2206(2HCl) + THP‐1 + TNF‐α groups were significantly decreased compared with the THP‐1 + TNF‐α group (*p* < .05). As shown in Figure 5C, it can be seen that the expression of p‐AKT and TTR protein was significantly decreased in HCCC9810 cells in the LY3214996 + THP‐1 + TNF‐α group compared with the THP‐1 + TNF‐α group; and the expression of NF‐κB and TTR protein was significantly reduced in the MK‐2206(2HCl) + THP‐1 + TNF‐α group (*p* < .05). This result indicates that LY3214996 and MK‐2206(2HCl) can inhibit TTR expression by inhibiting ERK and AKT under the action of TNF‐α + macrophages and are related to the NF‐κB pathway.

## 4 DISCUSSION

cHCC‐CCA is a heterogeneous tumor containing both HCC and iCCA components. According to the latest WHO classification, cholangiocarcinoma containing hepatocellular differentiation is classified as cHCC‐CCA, while in the absence of hepatocellular differentiation, it is classified as tubular iCCA.^22^ We performed differential gene analysis of cHCC‐CCA and CCA through the GEO database and showed that FGFR2 was highly expressed in the CCA group, while the expression of TTR was elevated in cHCC‐CCA. WGCNA also verified the correlation between TTR and cHCC‐CCA. It has been shown that the genetics of classic cHCC‐CCA is similar to that of HCC but different from iCCA.^23^

In addition to being a carrier, TTR is also widely recognized for its close association with amyloidosis. However, studies on the mechanism of action of TTR in tumors are scarce. Studies have shown that TTR increases lung tumor cell proliferation both in vitro and in vivo by stimulating capillary‐like tube formation and increasing endothelial cell proliferation through AKT.^19^ Zhang et al.^18^ found that elevated TTR expression in prostate cancer patients was closely associated with the risk of disease progression. Also, overexpression of TTR may be related to enhanced anoikis resistance and metastatic potential of prostate cancer cells. Our results showed that TTR was highly expressed in HCCC9810 compared to huh‐28 and positively correlated with the invasive ability of hepatobiliary tract cancer cells. In addition, TTR interacts with TAMs and affects the migration and invasive ability of the hepatocellular cholangiocarcinoma cells.

We induced THP‐1 polarization toward M1‐like macrophages by TNF‐α and subsequently co‐cultured with HCCC9810 to mimic the tumor microenvironment. We found that the invasive ability of HCCC9810 was enhanced in the THP‐1 + TNF‐α group, while in the TNF‐α group, there was no significant change in invasive ability. Xiao et al.^14^ found that M1‐like macrophages up‐regulated MME and MMP14 expression in oral squamous cell carcinoma cells, thereby promoting the epithelial‐mesenchymal transition (EMT) process and inducing cancer stem cell‐like cell formation (CSCs). This process is achieved through an IL6/Stat3/THBS1 feedback loop. Oshi and colleagues' transcriptomic analysis of breast cancer revealed that high M1 macrophage tumors and high M1/M2 ratio tumors did not provide an increased survival benefit, and those high M1 macrophage tumors were associated with clinically aggressive features.^24^ Such results are quite different from the current findings on the effects of TAMs on tumor invasion and metastasis. These findings suggest that TAMs of the M2 phenotype play an essential role in EMT and tumor metastasis.^25^ We suggest that the effects of TAMs in EMT and tumor metastasis are not dominated by M1‐ or M2‐polarized macrophages alone. There is a mixed state of M1‐like and M2‐like macrophages in the tumor microenvironment.^26^ Penny and colleagues also reported that pancreatic ductal adenocarcinoma produced TAMs expressing both M1 (IL‐1β, IL6, and TNF‐α) and M2 (CD163, CD206, and Arg1) markers.^27^ Through in vitro co‐culture and in vivo experiments, Wei and colleagues also demonstrated that in the presence of mixed phenotype‐TAMs, colorectal cancer cell migration, invasion, and metastasis were accompanied by increased EMT phenotype.^28^ In addition, the effect of TAMs on tumor cell invasion is also related to molecules secreted by the tumor cells themselves. We found that TTR had a promotive effect on the invasive ability of hepatobiliary tumor cells under co‐culture conditions with TAMs. Recently, it has been shown that THBS1‐containing exosomes activate M1‐like macrophages, which in turn promote the malignant migration of oral squamous cell carcinoma.^29^ This crosstalk of TAMs with tumor cells may be achieved through paracrine signaling.

Crosstalk of three signaling pathways, NF‐κB, ERK, and AKT, affects the invasive ability of cHCC‐CCA. In our study, we found that under the combined effect of TAMs and TNF‐α, the expression of NF‐κB, p‐AKT, and p‐ERK in HCCC9810 was significantly reduced, accompanied by a decrease in the invasive ability of HCCC9810. However, the addition of recombinant TTR enhanced the invasive ability of cancer cells instead, accompanied by elevated NF‐κB, p‐AKT, and p‐ERK protein expression. At the same time, we added ERK and Akt‐specific inhibitors. We found that protein expression of TTR and NF‐κB subsequently decreased, accompanied by a weakening of cancer cell invasion. The NF‐κB signaling pathway regulates the expression of EMT‐TFs, driving the EMT process and increasing tumor metastasis. Theabrownin inhibited NF‐κB signaling to reduce the expression levels of ZEB1, Slug, and Snail1 expression levels, increase E‐cadherin levels, and inhibit EMT, facilitating inhibition of osteosarcoma migration.^30^ PI3K/Akt is an essential upstream mediator of NF‐κB.^31^ AKR1B10 induces PI3K/Akt signaling, stimulates the NF‐κB pathway, promotes the expression levels of ZEB1, Slug, and Twist in EMT induction, and increases the migration rate of breast tumor cells.^32^ ERK1/2 is the mitogen‐activated protein kinases (MAPK) signaling pathway. The most critical members During metastasis, MAPKs regulate EMT, invasion, angiogenesis, and resistance to therapy.^33^ In highly invasive triple‐negative breast cancer cells, inhibition of TGF‐β blocks the ERK/NF‐κB/Snail signaling cascade, helping to restore the epithelial phenotype and reduce cell migration.^34^

This study found that TTR promotes the invasive ability of hepatobiliary cell carcinoma cells and is associated with M1‐like TAMs through in vitro cellular assays. Of course, there are shortcomings in our study: first, we lacked clinical samples to confirm the expression of TTR in cHCC‐CCA and the relationship between TTR and prognosis. Second, we constructed a tumor microenvironment with only single immune cells and cytokines, while it was not clarified whether TAMs phenotypes were changed in the co‐culture system. Third, we lacked in vivo experiments to validate further the relationship between hepatobiliary cell carcinoma and TAMs, which needs to be further confirmed by follow‐up studies.

## 5 CONCLUSIONS

In summary, this study found that TTR expression was increased in cHCC‐CCA compared with CCA and correlated with the invasion of HCCC9810. TNF‐α induced polarization of THP‐1 to M1‐like macrophages after co‐culture with HCCC9810 and invasion of HCCC9810 cells was significantly increased. This change in invasion capacity was positively correlated with TTR. This effect was achieved by activating AKT/NF‐κB and ERK signaling pathways, promoting EMT. TTR may be a new target for the treatment of unresectable cHCC‐CCA. It is concluded that TTR can promote cHCC‐CCA invasion by regulating AKT/NF‐κB and ERK signaling with the assistance of M1‐like TAMs.

## AUTHOR CONTRIBUTIONS

**Kun Ke:** Conceptualization (equal); project administration (equal); writing – original draft (equal). **Junqing Lin:** Methodology (equal); validation (equal); writing – original draft (equal). **Ning Huang:** Validation (equal). **Leye Yan:** Investigation (equal); methodology (equal). **Rihua Liao:** Investigation (equal). **Weizhu Yang:** Conceptualization (equal); writing – review and editing (equal).

## CONFLICT OF INTEREST STATEMENT

The authors have stated explicitly that there are no conflicts of interest in connection with this article.

## ETHICS STATEMENT

Not applicable.

## Supporting information


**Table S1.** The results of the GO analysis.

**Table S2.** The results of the KEGG analysis.

**Figure S1.** Detection of HCCC9810 cell viability by CCK‐8 after treating HCCC9810 with different concentrations of TNF‐α (*p < .05 vs. Control).

## Data Availability

The data sets generated and analyzed during the current study are available in the Gene Expression Omnibus (GEO) database (https://www.ncbi.nlm.nih.gov/geo/). All data analyzed during this study are included in this published article and its supplementary information files.
