# Supplementary figures and images for: Transthyretin promotes the invasion of combined hepatocellular cholangiocarcinoma by tumor‐associated macrophages
Source: Cancer Rep (Hoboken). 2023 Sep 9;6(10):e1888. doi: 10.1002/cnr2.1888 (PMC10598247; doi:10.1002/cnr2.1888)

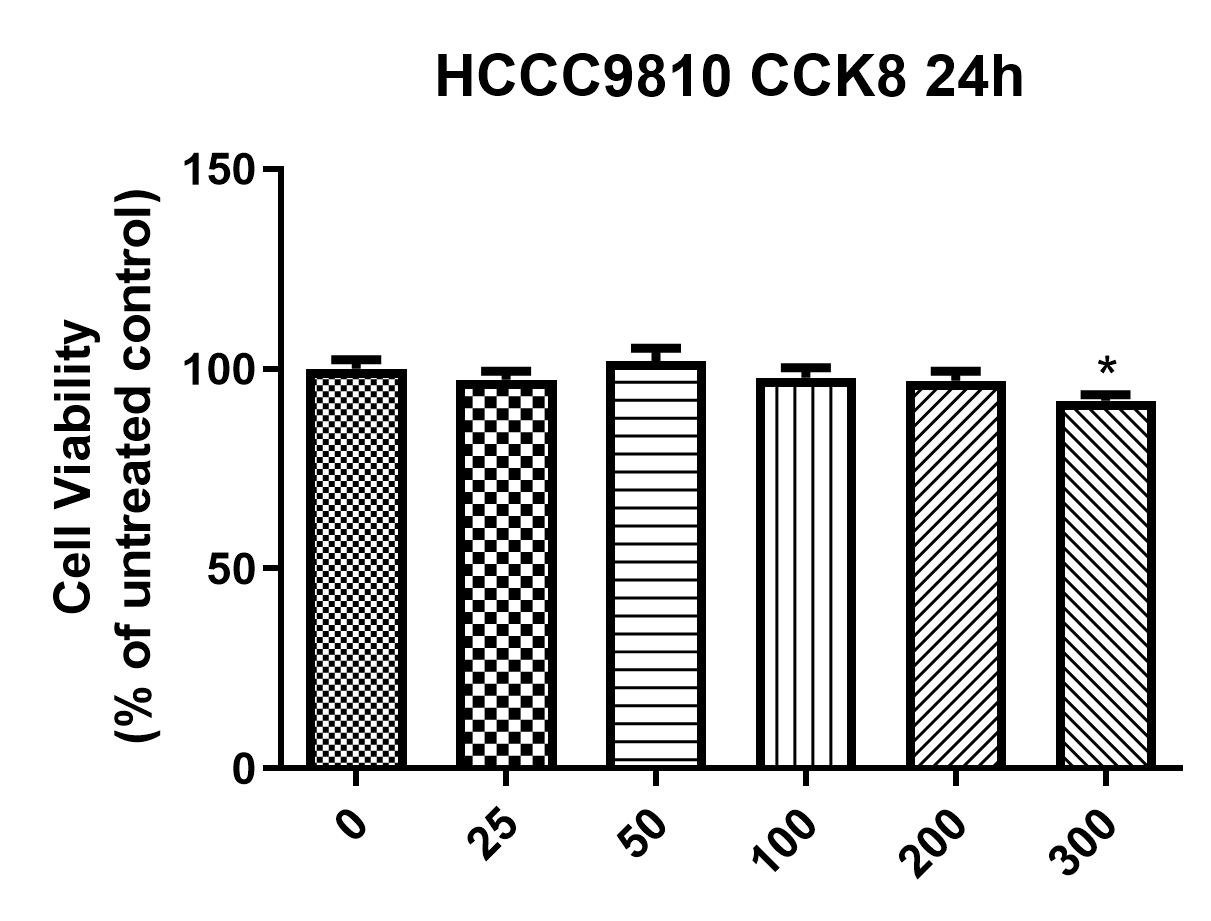

Supplement: Supplementary file 3 — Figure S1. Detection of HCCC9810 cell viability by CCK‐8 after treating HCCC9810 with different concentrations of TNF‐α (*p < .05 vs. Control). [file CNR2-6-e1888-s002.tif]
